# Comparison of Physiological Stress Indices in Anesthetized and Manually Restrained Leopard Sharks, *Triakis semifasciata*

**DOI:** 10.3390/biology13110878

**Published:** 2024-10-29

**Authors:** Meghan M. Holst, Catharine J. Wheaton, Alexandra N. Schoen, Jill V. Spangenberg, Kevin T. McEligot, Melissa L. Schouest, Charlene M. Burns, Natalie D. Mylniczenko

**Affiliations:** 1 Aquatic Health Program, School of Veterinary Medicine, Department of Anatomy, Physiology, and Cell Biology, University of California, One Shields Avenue, Davis, CA 95616, USA; 2 Disney’s Animals, Science and Environment, Disney’s Animal Kingdom^®^ and the Seas with Nemo and Friends^®^, Lake Buena Vista, FL 32830, USA; catharine.j.wheaton@disney.com (C.J.W.); charlene.m.burns@disney.com (C.M.B.); natalie.mylniczenko@disney.com (N.D.M.); 3 Department of Biology, University of Winnipeg, Winnipeg, MB R3B SE9, Canada; a.schoen@uwinnipeg.ca; 4 Animal Care Department, Aquarium of the Bay, San Francisco, CA 94133, USA; jvspangenberg@gmail.com (J.V.S.); kevin@bay.org (K.T.M.); melissa@bay.org (M.L.S.)

**Keywords:** 1α-hydroxycorticosterone, health assessment, physiological stress, anesthesia, elasmobranch

## Abstract

Background: Leopard sharks (*Triakis semifasciata*) are abundant, coastal, eastern Pacific, mesopredatory sharks and are frequently managed in aquariums and zoos. Medical examinations are a routine part of good husbandry practices, but the handling protocols vary greatly between facilities. In this study, we compared the physiological stress responses of *T. semifasciata* associated with manual restraint and sedated handling under parallel holding and handling procedures in a 10 min interval. Methods: Blood was collected and analyzed for the hematocrit (Hct), lactate, glucose, beta-hydroxybutyrate (β-HB), pH, blood gasses, 1α-hydroxycorticosterone (1α-OHB), and corticosterone (B). Results: Overall, a minimal physiological stress response was observed in both groups. However, statistically significant increases in the PCO_2_, β-HB and lactate occurred, while the pH_TC_, SO_2_, and BE decreased over time in the manual-restraint group. In the sedated group, the B, PCO_2_, and lactate increased while the pH_TC_, SO_2_, and base excess (BE) decreased over time. When comparing treatments, the sedated group had a higher B and PCO_2 TC_ and a lower pH_TC_, BE, and SO_2_ than the manual-restraint group at the second time point. Conclusions: This study indicates that manual restraint is comparable to the described sedation in *T. semifasciata* for minimally invasive procedures. The overall results show that this species had low physiological stress responses to both methods.

## Simple Summary

Leopard sharks (*Triakis semifasciata*) are abundant, coastal, eastern Pacific, mesopredatory sharks and are frequently managed in aquariums and zoos. Medical examinations are a routine part of good husbandry practices, but the handling protocols vary greatly between facilities. In this study, we compared the physiological stress responses of *T. semifasciata* associated with manual restraint and sedation under parallel holding and handling procedures in a 10 min interval. Blood was collected and analyzed for common physiological measurements used in shark and ray stress studies. Overall, a negligible stress response was observed in both experimental groups. This study indicates that manual restraint is comparable to the described sedation in *T. semifasciata* for minimally invasive procedures. The overall results show that this species had low stress responses to both methods.

## 1. Introduction

The handling of sharks is a common practice for field research, fisheries, and aquarium animal management. However, handling techniques will vary greatly depending on the species, staff training, and the handling purpose. Anesthesia is often used under managed care to reduce the perceived animal stress and facilitate the safety of both the animal and the care staff during handling procedures [1,2,3,4]. Manual restraint is almost exclusively used in field research, as wild sharks are often too large to reasonably anesthetize, monitor, and allow to recover fully before their release into the wild. In field settings, it is also essential to minimize stress by keeping the sampling times as short as possible [5]. Additionally, some anesthetics may require days to weeks of withdrawal from tissues and should not be used for animals that will be released back into the wild and potentially recaptured as food [1].

Under any application, critically evaluating the handling protocols is necessary for optimizing animal care, particularly in controlled environments, when both manual restraint and anesthesia are readily available techniques. Evaluating the physiological response that animals have to these techniques can serve as a proxy for how well an experience is tolerated. The stress axis should, therefore, be measured under conditions of interest to critically evaluate the impact of different handling protocols. The primary, secondary, and tertiary stress responses have been well documented in teleosts and elasmobranchs [6,7,8,9,10]. The primary stress response encompasses the brain’s initial perception of a stressor, with the subsequent release of catecholamines (epinephrine and norepinephrine) and an initial release of energy metabolites [10]. The secondary stress response is a later response (but within minutes of the stressor) with the release of corticosteroids, which can impact osmoregulation and the mineral balance [10]. The tertiary stress response involves the whole organism over a longer time period, where the stressor is seen to impact or alter the organism’s behavior, immune response, disease resistance, growth, reproduction, or overall biological fitness [6,9,11,12].

Animal care staff sometimes suggest using anesthesia or analgesics to ensure animal well-being during research or clinical procedures [3]. While anesthesia can minimize the handling stress, studies evaluating its physiological effects in elasmobranchs remain limited. For instance, Frick et al. (2009) found that sedation using isoeugenol anesthesia (AQUI-S^®^) increased the plasma lactate and potassium concentrations in Australian swell sharks compared to a non-sedated control group, although the plasma glucose remained unaffected [13]. These findings highlight the species-specific nature of stress responses and suggest that factors like the lack of appropriate ventilation and the capture methods may influence the results. Other studies, such as one on Atlantic sharpnose sharks, have reported reduced plasma lactate, glucose, and hematocrit levels after handling when using isoeugenol [14]. However, no research has compared the physiological responses between manual restraint and sedation in elasmobranchs to determine the most effective technique for minimizing stress.

The glucose, lactate, and pH are some of the most common and leading measurements used to identify physiological stress in elasmobranchs, particularly for field research [11,12,13,14]. Other tests can serve a function in the assessment of stress, including the hematocrit (Hct), osmolality (osmo), and beta-hydroxybutyrate (β-HB). The hematocrit can change in teleosts and elasmobranchs with stress or exercise, although this may be species-dependent [15]. The osmolality serves as an indicator of health and fluid balance, with changes under stress suggested to result from the role of mineralocorticoids, which are associated with stress hormones in elasmobranchs [16]. While the β-HB may not be directly linked to stress-induced energy mobilization, it is a valuable marker of an animal’s initial health status and liver condition [17,18]. Therefore, it provides important context for understanding an animal’s overall physiological state, complementing glucose, osmolality, and lactate measurements to assess how the animal was handled and its broader health condition. Beyond this and basic blood work for health assessments, hormones related to the mobilization of energy during physiological stress can significantly improve our understanding of an animal’s response to its environment. The endocrine response in ray-finned fishes mobilizes cortisol as the primary glucocorticoid during stressful events [10]. In elasmobranchs, the dominant circulating corticosteroid is 1α-hydroxycorticosterone (1α-OHB) [16,19,20,21,22]. Iki et al. (2020) demonstrated an increase in the 1α-OHB concentrations associated with a stressor in elasmobranchs. They measured 1α-OHB and its theorized precursor, corticosterone (B), to evaluate the effect of the treatment conditions on the circulating concentrations of these corticosteroids [22]. While B is the equivocal precursor, this has still not been demonstrated experimentally.

In the present study, we compared the physiological stress response of anesthetized and manually restrained leopard sharks (*Triakis semifasciata*) during basic physical examinations with a commonly used sedative, tricaine methanesulfonate (MS-222, Syndel, Washington, DC, USA). *T. semifasciata* sharks are common elasmobranchs in aquariums, with the capacity for tolerating a variety of temperatures [23] and salinity ranges [24], which lends them to be a hardy species under managed care [25,26]. While MS-222 may mitigate the potential stress or injury due to handling resistance in many fish species, it also has been shown to cause an aversive response [26,27,28]. We hypothesized that the anesthetized treatment group could induce a stronger physiological response than manually restrained fish for the same procedure. This stems from the understanding that the systemic effects of chemical exposure impact behavior and metabolism [29,30,31], potentially causing more physiological stress than manually restraining an animal while it is awake for a basic procedure. Given the limited literature definitively showing that either chemical or mechanical restraint consistently causes more stress across species [13,14], we aimed to test this hypothesis in a common husbandry procedure in elasmobranchs under managed care to contribute data that clarify the relative impacts of sedation versus manual restraint. This was tested by sampling blood at the earliest possible time point and then at the end of the procedure, and measuring the physiological changes in the plasma. The blood Hct, point-of-care (POC) analytes (blood gasses and associated values), glucose (Glu), lactate, osmo, β-HB, and corticosteroids 1α-OHB and B were measured to critically compare the two sampling methods. These tests help reflect the physiological shifts that occur with physical handling, hypoventilation, and known stressors as part of the endocrine response. Here, changes in these indices will help determine if either methodology produces a change in the acute stress-axis response. 

## 2. Materials and Methods

### 2.1. Population Demographics and Health

The study population was 16 adults (*T. semifasciata*) of unknown ages, consisting of 4 males, 12 females, and 0 individuals of an unknown sex (notation: 4.12.0; 94–160 cm total lengths (TLs)), and 6 juvenile sharks (1 male, 5 females, and 0 of an unknown sex; notation: 1.5.0; 61–68 cm TLs). The juvenile sharks were 17 months old, born under human care, and likely as habituated to handling as the adults, which had all remained in the exhibit for at least 16 months before the time of this study. All the animals were housed in enclosures at the Aquarium of the Bay in San Francisco, CA, USA. Husbandry standards were maintained as outlined by the Association of Zoos and Aquariums (AZA) accreditation standards. The animals were housed in two primary habitats at the facility: The Sharks of Alcatraz tunnel (SAT; ~350,000 gallon) and a holding enclosure (HLD; ~960 gallons). All the animals were rotated routinely into the aquarium’s elasmobranch touch pool, but not in the immediate 30d prior to this study. Natural seawater was pumped from San Francisco Bay and filtered through industrial sand filtration, ultra-violet sterilization, and ozonation before reaching the enclosures. The water temperature remained between 14.15 and 15.5 °C, the pH was between 7.34 and 7.52, the dissolved oxygen (DO) was between 104 and 106%, and the salinity was between 29.3 and 29.6 ppt salinity for the duration of the experimentation in both enclosures. The water quality (pH, temperature, salinity, and dissolved oxygen) maintained throughout the experiment was considered to be within normal limits [27].

The animals’ health was closely monitored by biologists who conducted daily assessments of their overall condition, including their behavior, activity levels, and appetite. All the experimental animals were provided the same food. Additionally, our staff veterinarian performed weekly health checks and administered annual physical exams to all the elasmobranchs within the study population. These exams included thorough evaluations of their body condition and overall health parameters. Based on these regular assessments, the veterinarian concluded that all the animals were in good physical health prior to and throughout the duration of the study. 

### 2.2. Experimental Groups

Both the SAT and HLD populations were divided into two experimental groups: sedated (*n* = 10) and manually restrained (*n* = 12). The population was biased towards females (5.17.0; Table 1). The 5 males in the SAT population were divided into each experimental group, while the juvenile male in the holding enclosure was randomly allocated to one of the experimental groups (Table 1). 

### 2.3. Handling Techniques 

All the juveniles in the HLD were netted from the surface using a rubberized soft net within 10 s of the first attempt, as all the animals were readily accessible in the HLD (~1 m deep). Individual juveniles were targeted first based on the ease of identification and access and were moved to a replicate holding enclosure after the procedure to reduce tank-mate-induced stress from prolonged netting activity in the enclosure. A maximum of two animals were sampled per day to reduce the degree of stress that may have occurred from exhibit disturbance. 

The adults were also netted from the surface when possible, from the SAT. However, due to the depth of the SAT (~3 m deep), it was not possible to net all 16 adults opportunistically from the surface. Therefore, aquarium divers entered the SAT to retrieve the remaining adults using rubberized nets for transport immediately to the surface for blood sampling. All the *T. semifasciata* sharks were handled by experienced shark-handling biologists one at a time for blood sampling. A maximum of four animals were sampled per study day to reduce the degree of stress that may have occurred from exhibit disturbance, similarly to the HLD capture procedures. 

### 2.4. Blood Sampling

**Figure 1:**
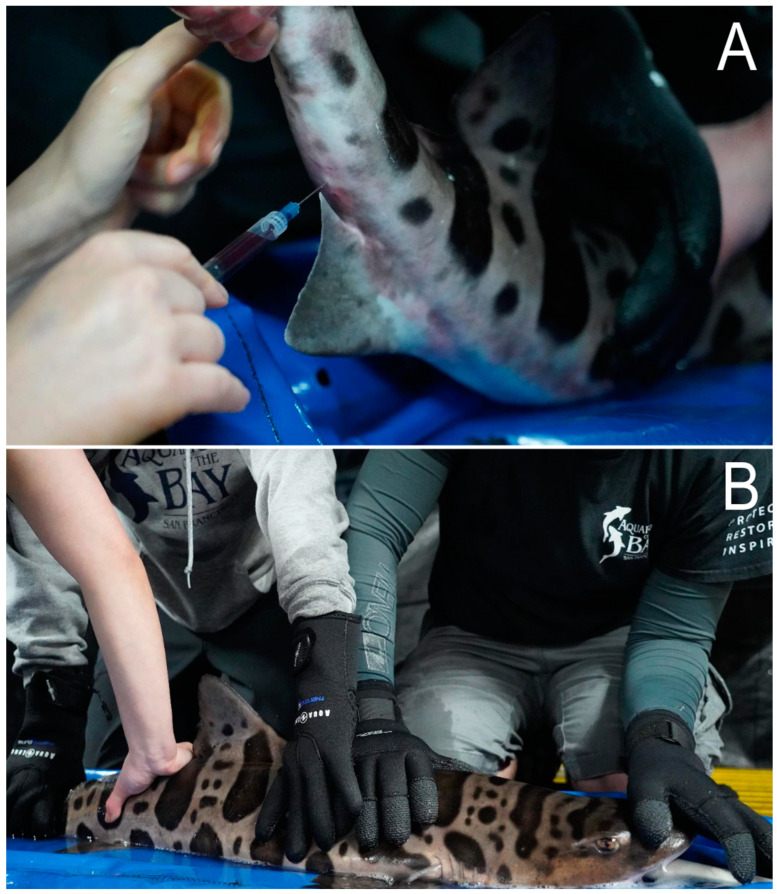
(**A**) A 2 mL blood sample was collected via caudal venipuncture ventrally by having restrainers lift the tail of the leopard shark (*Triakis semifasciata*). (**B**) There were 3 to 4 restrainers per animal to ensure the safety of the animal and staff. One person was responsible for keeping the manifold in place so that seawater effectively flushed water over the gills during the entire procedure. The remaining staff restrained the animal near the pectoral and anal fins to reduce movement and potential injury.

Sampling was performed for routine health assessments. Once an animal was removed from an enclosure, it was manually restrained on a mat adjacent to its holding enclosure to obtain an immediate ~2 mL blood sample (first sample; time = 0 min). Blood was taken via the ventro-caudal tail vessel, immediately posterior to the anal fin, with a 2” 22-gauge needle (Figure 1A). All the initial blood samples were taken within 2 min from the point when the animal made contact with a net. Most samples were presumed to be arterial based on the anatomy and relative oxygen trends, although the mixing of samples was possible given the proximity of the vessels [32].

After the first blood sample, the animals were either maintained in manual restraint or placed into anesthesia for the sedated experimental group. The sedated experimental group was placed in a holding container (804 L of saltwater). Supplemental pure oxygen was administered through an air stone and monitored to maintain 100–120% mg·dL^−1^ dissolved oxygen (DO) in the water. In the sedated experimental group, MS-222 (80 mg·L^−1^) was added to the holding container to provide sedation, and sodium bicarbonate (NaHCO_3_) was added at a 2:1 ratio to maintain a stable pH before each animal was transferred [28] to the bath for immobilization. The water pH was monitored and remained between 7.52 and 7.56 throughout the study. After 10 min, a second blood sample was collected from each fish. In the manual-restraint experimental group, the sharks were manually restrained for 10 min with untreated exhibit water continuously flushing over their gills, and a second blood draw was taken 10 min after the first blood sample for a matched time point comparison with the sedated group (Figure 1B). In both treatment groups, the animals were ventilated with water continuously over the gills. The morphometrics were collected by a second biologist between the first and second blood samples as part of their routine yearly physical examinations. 

### 2.5. Animal Recovery

The manually restrained animals in both exhibits were immediately returned to their respective exhibits following the second blood sample. The sedated animals were held by the animal care staff in untreated saltwater until they were able to orient themselves and swim independently. All the animals remained in either a soft, rubberized net (juvenile animals) or in a medical pool (adult animals) until they fully recovered from sedation. 

### 2.6. Blood Processing

For each animal and time point, 0.1 mL aliquots of whole blood were analyzed with a point-of-care (POC) device (Abbot i-STAT Alinity; Heska Corp., Fort Collins, CO, USA) for blood gasses and pH using a CG8+ cartridge (see Table 2). Potassium, sodium, Hct, calcium, and hemoglobin were not able to be read in the POC unit, and therefore, they were not used. The temperature correction (TC) was adjusted based on the environmental temperature at the time of sampling. These samples were processed immediately after collection (<1 min). The remaining whole blood was then transferred to a microcentrifuge tube containing lithium heparin. Heparinized blood was then drawn into two capillary tubes and centrifuged at 12,600× *g* for three minutes for the determination of the Hct values (TRIAC^TM^ Clay Adams^®^ centrifuge, Adams Manufacturing, Portersville, PA USA). The remainder of the sample was spun at 1500× *g* for five minutes. The plasma was then decanted into clean microcentrifuge tubes and stored at −20 °C for a later analysis. 

All the samples for these parameters used thawed banked plasma. Glucose was run using 5 µL of the sample and was analyzed in an Accu-Chek^®^ unit (Roche Diabetes Care, Inc., Indianapolis, IN, USA). Lactate was run using 5 µL of the sample and was analyzed in a Lactate Scout^®^ unit (EKF diagnostics, Boerne, TX, USA). Osmolality was run using 10 µL of the sample and was analyzed in an Osmette^®^ III (Precision Systems, Natick, MA, USA). β-HB was run using 5 µL of the sample and was analyzed in a STAT-Site^®^ M (EKF diagnostics, Boerne, TX, USA).

### 2.7. 1α-Hydroxycorticosterone and Corticosterone Enzyme Immunoassays

The serum extraction, processing, and enzyme immunoassays (EIAs) were performed at Disney’s Animal Kingdom^Ⓡ^ Science Center lab (Orlando, FL, USA) by closely following the methods described by Schoen, Treberg, et al. (2021); Schoen, Bouyoucos, et al. (2021); and Wheaton et al. (2018) [8,20,21]. Briefly, 0.5 mL plasma samples were extracted in 1.5× volume ice-cold acetonitrile. After centrifugation, the supernatants were evaporated to dryness at 35 °C (Labcono^®^ RapidVap^®^, Kansas City, MO, USA) [20] and the samples were then reconstituted in an assay buffer such as to be appropriately concentrated (1α-OHB) or diluted (B) to remain in the readable range of the in-house assay standard curves [21]. Samples with B concentrations below the detectable limit of the assay were reported as having a zero concentration. All the chemicals (purchased from Sigma Aldrich, St. Louis, MO, USA) and equipment remained the same unless otherwise stated [8,20,21]. The samples were randomly assorted on the assay plates and respectively assayed in triplicate (1α-OHB) or duplicate (B).

### 2.8. Statistical Analysis

All the statistical analyses were conducted in R (version 1.3.1093) and statistical significance was accepted at α = 0.05. The datasets were subjected to Shapiro–Wilk’s and Levine’s tests to assess normality and equal variance. If the assumptions of these tests were not met, the data were transformed using rank transformations. The dataset for lactate did not pass the test for equal variance using standard methods of transformations, and thus, it was analyzed using a series of Student’s *t*-tests. The *t*-tests were performed between the restraint methods within the individual sampling time points, and between time points with the restraint methods. The resulting *p*-values from the *t*-tests were further subjected to a Bonferroni correction to account for the repeated analysis.

The analyses for all the other datasets were similar to the method used by Schoen, Treberg, et al. (2021), with some alterations [20]. Two-way linear mixed-effects models (‘lmer’ function in ‘lme4’ package) [33] were used to model the significance of the blood sampling time, the restraint method, and the interaction between these factors. Shark identification was added to each model as a random variable to account for non-independence and repeat sampling [34]. The significance in the linear mixed-effects models (of each factor individually or the interaction between factors) was further analyzed using the estimated marginal means method (‘eemeans’ function in the ’eemeans’ package) [35]. Finally, a correlation analysis between the datasets was performed using Pearson’s product-moment correlation coefficients (*r*).

## 3. Results

### 3.1. Corticosteroid and Energy Metabolite Changes

**Figure 2:**
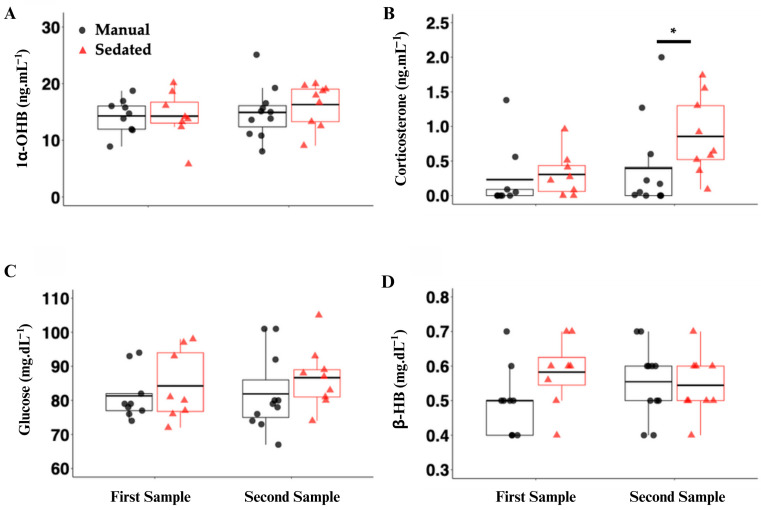
Plasma analysis of corticosteroids ((**A**) 1α-hydroxycorticosterone (1α-OHB, ng·mL^−1^); (**B**) corticosterone (B, ng·mL^−1^)) and energy metabolites ((**C**) glucose (Glu, mg·dL^−1^); (**D**) beta-hydroxybutyrate (β-HB, mg·dL^−1^)) in manual-restraint (black circles) and sedated (red triangles) leopard sharks (*Triakis semifasciata*). Black horizontal lines within the boxplots represent the mean. Asterisks (*) and black bars above the plot represent means that are significantly different between treatments at the 10 min sampling time point (treatment effect; *p* < 0.05).

There were no significant differences in the plasma 1α-OHB concentrations between the sampling times or the restraint treatment groups (Figure 2A). There was a significant increase in the plasma B concentration between the sampling times in the sedated treatment group (*p* < 0.001), as well as between the manual and sedated groups at the time of the second sampling time point (Figure 2B; *p* = 0.004). There were no significant differences in the plasma glucose concentration between the sampling time points in the manual-restraint or sedated treatment groups or between the treatment groups at the second sampling time point (Figure 2C). β-HB significantly increased from time zero to 10 min in the manual-restraint group only (Figure 2D; *p* = 0.015), but it was not different between groups at the time of the second sample.

### 3.2. Lactate, pH, and Base Excess Changes

**Figure 3:**
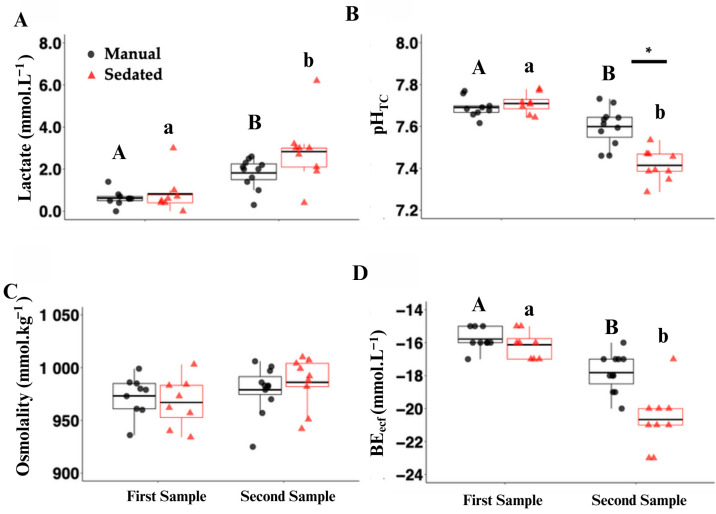
Plasma analysis of lactate ((**A**) mmol·L^−1^), pH_TC_ (temperature-corrected (TC); (**B**)), osmolality ((**C**) mmol·kg^−1^), and base excess ((**D**) BE_ecf_; mmol·L^−1^) in manual-restraint (black circles) and sedated (red triangles) leopard sharks (*Triakis semifasciata*). Black horizontal lines within the boxplots represent the mean. Asterisks (*) and black bars above the plot represent means that are significantly different between treatments at the 10 min sampling time point (treatment effect; *p* < 0.05). Capital letters indicate means that are significantly different within the manual-restraint treatment between the two sampling time points (first sample = time 0; second sample = 10 min; *p* < 0.05). Lowercase letters indicate means that are significantly different between the two time points within the sedated treatment (*p* < 0.05).

The plasma lactate concentrations significantly increased (all *p* < 0.001), while the plasma pH significantly decreased (all *p* < 0.007) between the sampling time points in both the manual and sedated treatment groups (Figure 3A,B). The plasma pH was also significantly lower in the manual-restraint group than the sedated group at the second time point (Figure 3B; *p* < 0.001). There were no significant differences in the osmolality between treatment groups or time points (Figure 3C). The base excess (BE) significantly decreased over time in both the manual and sedated treatment groups (Figure 3D; *p* < 0.001) and was significantly lower in the sedated group, as compared to the manual-restraint group, at the second time point (Figure 3D; *p* < 0.001).

### 3.3. Respiratory and Osmolality Changes

**Figure 4:**
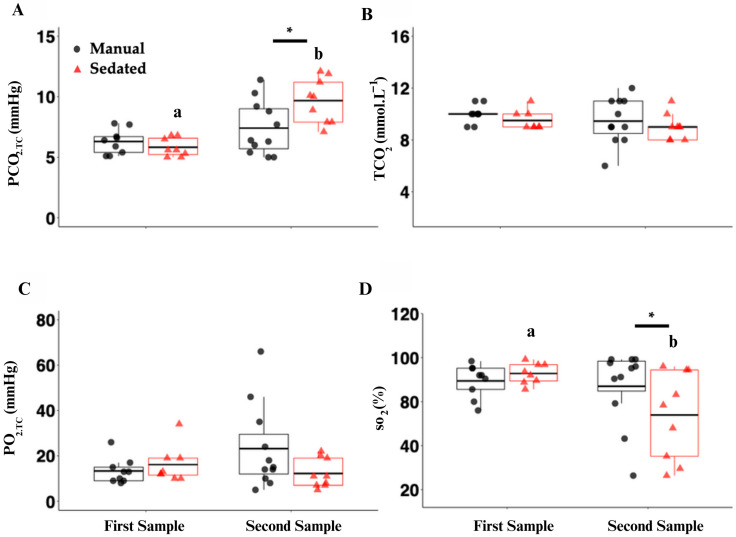
Plasma analysis of PCO_2.TC_ (partial pressure of carbon dioxide, temperature-corrected (TC); (**A**), mmHg); TCO_2_ (total carbon dioxide; (**B**), mmol·L^−1^); PO_2.TC_ (partial pressure of oxygen; (**C**), mmHg); and SO_2_ (oxygen saturation; (**D**), %) in manual-restraint (black circles) and sedated (red triangles) sharks. Black horizontal lines within the boxplots represent the mean. Asterisks (*) and black bars above the plot represent means that are significantly different between treatments at the 10 min sampling time point (treatment effect; *p* < 0.05). Lowercase letters indicate means that are significantly different between the two time points (first sample = time 0; second sample = 10 min) within the sedated treatment (*p* < 0.05).

The whole-blood PCO_2.TC_ significantly increased in the sedated group between time points (*p* < 0.001) and was significantly higher in the sedated treatment group than the manual group at the second time point (Figure 4A; *p* = 0.004). There were no significant differences in the total carbon dioxide (TCO_2_; Figure 4B) or partial pressure of oxygen (PO_2.TC_; Figure 4C) between the time points or treatment groups. Lastly, the saturated oxygen (SO_2_) showed a weak significant decrease in the sedated group between time points (*p* = 0.044), with a significant difference between treatment groups at the second time point (Figure 4D; *p* = 0.040).

### 3.4. Correlations Between Physiological Parameters

**Figure 5:**
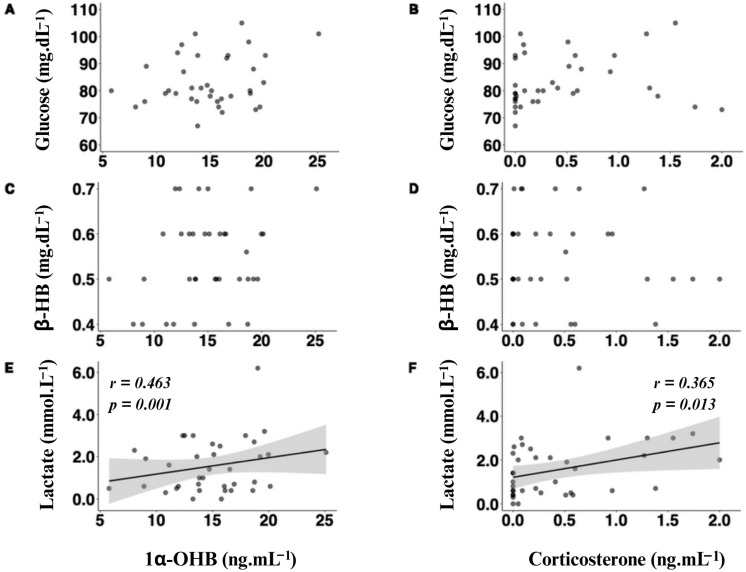
Pearson’s product-moment correlation coefficient plots, depicting the values of the plasma energy metabolites glucose (Glu; (**A**,**B**), mg·dL^−1^), beta-hydroxybutyrate (β-HB; (**C**,**D**), mg·dL^−1^), and lactate ((**E**,**F**), mmol·L^−1^), evaluated against the measured corticosteroids 1α-hydroxycorticosterone (1α-OHB; (**A**,**C**,**E**), ng·mL^−1^) and corticosterone (B; (**B**,**D**,**F**), ng·mL^−1^).

There were no significant correlations between Glu and either 1α-OHB or corticosterone (Figure 5A,B). There was no correlation between β-HB and 1α-OHB or corticosterone (Figure 5C,D). There was, however, a significantly positive correlation between lactate and 1α-OHB a (Figure 5E, *p* = 0.001), as well as lactate and corticosterone (Figure 5F, *p* = 0.013) and lactate and whole-blood pH_TC_ (*p* < 0.0001; r = −0.7508). There was a trending correlation between plasma corticosterone and 1α-OHB concentrations, although this was not significant (*p* = 0.058; r = 0.454).

## 4. Discussion

### 4.1. Summary

In this study, we aimed to compare manual restraint and sedation to identify any differences in the physiological stress responses of the leopard shark, *Triakis semifasciata*. The treatment period was brief (<10 min), but mimicked a typical procedure at the facility. Our findings indicate that both treatment groups exhibited minimal and biologically insignificant changes in their blood physiology, indicating that neither method compromised the overall health of the sharks. While some results in the manual-restraint treatment exhibited a slightly lower (but statistically significant) change in physiology during their physical examination, neither group demonstrated physiological changes that would be considered to be of great concern based on our current understanding of elasmobranch health [36]. This may have been due to several factors, including species durability, the high level of animal handling experience of the staff, and *T. semifasciata*’s habituation to yearly physical examinations and rotation into the aquarium’s elasmobranch touch pool, during which they are routinely removed from their habitat.

### 4.2. Physiological Changes of Both Treatment Groups

#### 4.2.1. Lactate and pH

The increase in lactate between the time points is consistent with other elasmobranch physiology studies, which have demonstrated that, during excessive muscle exertion, hypoventilation, or physiological stress, lactate increases [11,12,37,38,39,40]. This is further supported by a significant decrease in the pH between the time points. In its normal response to reduce pH changes, the body uses bicarbonate to buffer the blood for pH recovery; thus, a homeostatic shift of HCO_3_ will occur and result, in part, in a concomitant change in the BE (the total of all the bases in the blood) as a response [41]. The oxygen saturation in general in point-of-care blood gas units is considered to be inaccurate due to difficulty in obtaining an arterial versus venous sample, and it is often highly variable; thus, the results are difficult to interpret [38].

Lactate can increase over extended periods or in response to significant stress or prolonged exertion. However, in this study and under this time frame, that was unlikely, as the baseline lactate levels were low and other physiological parameters remained within acceptable ranges. Greater, but biologically insignificant, changes occurred in the sedated treatment group, likely due to the exposure to MS-222, as well as the excitatory phase that elasmobranchs experience as they undergo anesthetic induction [28]. There is no physiologic difference in the Hct, Glu, osmo, or β-HB measurements between the manual and sedated group or between time points, supporting a low initial stress response.

#### 4.2.2. Corticosterone

Corticosterone is one of the traditional vertebrate steroid hormones associated with stress [42], but its role in the elasmobranch stress response has not yet been established. In the present study, a statistically significant, but physiologically small, increase in corticosterone was observed between the time points in the sedated group only. Additionally, the plasma corticosterone concentration was significantly greater in the sedated group as compared to the manual group within the second time point. While an increase in corticosterone may reveal changing physiological parameters over a 10 min period, other studies, such as that by Schoen, Treberg, et al. (2021), have not demonstrated the mobilization of energy metabolites after *S*. *suckleyi* was injected with corticosterone, and they exhibited a negative correlation between corticosterone and glucose [20]. However, this study began collecting blood from an indwelling cannula after either 24 h or 48 h after cannulation, and therefore, the glucose may have already increased and remained high from the cannulation procedure before the experimental blood draws were taken. While a statistically significant change in corticosterone was observed in this study, the values still remained biologically negligible when comparing the corticosterone values to other species that clearly demonstrate a treatment stress response (e.g., Schoen et al., 2021). However, this may potentially represent a small increase in corticosterone as a precursor to the production of 1α-OHB [20]. Further research is still needed to determine the role of corticosteroids in the elasmobranch stress response.

### 4.3. Treatment Group Variations 

The comparative blood results demonstrated a slight, but statistically significant, change for one or both treatment groups between the first and second time points for lactate, pH, TCO_2_, BE, SO_2_, and corticosterone (only in the sedated group). While some point-of-care blood gas measurements in elasmobranchs are not considered accurate [43,44], many values are appropriate for physiologic evaluations [45], particularly when evaluating trend differences. Some of these inaccuracies result because of animal temperature as well as algorithm differences within the unit that do not match elasmobranch biology. Despite this, the values that were obtained were useful within the obtained ranges to provide information on the status of the animal, as will be discussed.

Additionally, there was a statistically significant difference at the second time point between the treatment groups for pH, PCO_2_, BE, SO_2_, and B, with a larger change occurring in the sedated group. The plasma pH was also significantly lower in the sedated group compared to the manual group after 10 min, suggesting a greater relative physiological response in the sedated group. The significant decrease in pH was correlated with a significant increase in blood lactate.

### 4.4. 1α-OHB and Its Observed Role in the Physiological Stress Response

Our study did not demonstrate significant variance in the 1α-OHB values between time points or treatment groups. This and the relatively low concentrations of Glu suggest that it may have been a minimal physiological stress response to a small stress stimulus. These results are similar to those of other studies, which have demonstrated the same pattern in spiny dogfish, *Squalus suckleyi* [20], and juvenile blacktip reef sharks, *Carcharhinus melanopterus* [8]. *T. semifasciata* demonstrated an overall low stress response compared to other species (e.g., great hammerheads, *Sphyrna mokarran*) [38,46]. Lactate is significantly correlated with 1α-OHB, as well as corticosterone. Lactate is expected to increase under these conditions because the animal is under anaerobic metabolism and some inevitable muscle exertion. 1α-OHB has been shown to correlate with lactate [46], but this relationship is underrepresented in the literature and needs further documentation with relation to 1α-OHB and B. Overall, without evidence of a clear, strong physiological stress response, it is difficult to evaluate the role of 1α-OHB as a stress hormone in our study, and therefore, we were unable to use it to definitely evaluate between manual restraint and sedation under these conditions.

### 4.5. Species-Specific Stress Response

The increase in lactate between the time points is consistent with other elasmobranch physiology studies, which have demonstrated that, when elasmobranchs experience strenuous exercise (muscle exertion), hypoventilation, or physiological stress, lactate increases [11,12,37,38,39,40]. This is further supported by a significant decrease in the pH between the time points. In the normal response to lower pH changes, the body uses bicarbonate to buffer the blood for pH recovery; thus, a homeostatic shift in HCO_3_ will occur and result, in part, in a concomitant change in the BE (a total of all bases in the blood) as a response [41]. Oxygen saturation in general in this unit is considered to be inaccurate and highly variable; thus, the results are difficult to interpret [45].

While we certainly observed some mild acute changes typical of a stress response between the time points (e.g., increased lactates), these are also expected changes during physical exertion for elasmobranchs, and overall, both β-HB and glucose remained low or within the expected normal ranges [46] compared to elasmobranch studies that have observed a more severe and concerning stress response with greater exertion [38,45,47].

### 4.6. Limitations

There were several limitations of this study, including a limited sample size of leopard sharks available. Ideally, all the animals would have been roughly the same age, held within the same exhibit, and collected in the same manner throughout the experiment. Additionally, environmental disturbances that could cause physiological stress outside of our experimental conditions were not accounted for. Environmental disturbances that could have contributed to an alteration from homeostasis include competition from tank mates for space or food, or increased interactions due to breeding purposes. Our sample size did not allow for us to evaluate the physiological differences between subgroups, such as evaluating the effects of sex, age, or exhibit location on the physiological responses. An increased sample size would also dampen the variable effects of the treatment groups, such as the level of training of the individuals manually restraining an animal. Further, higher animal numbers would better account for individual shark physiological differences and differences in handling these sharks by trained professionals.

Comparing wild or naive *T. semifasciata* to managed care animals under identical handling events would further support whether the results seen in this study are due to the robustness of the species or the habituation of the individuals. Regardless, this study indicates that neither manual restraint nor sedation produces much change in the physiological stress response of *T. semifasciata* in this population during physical examinations. These results are likely species-specific and circumstance-specific, and should not be used to determine the best techniques in other elasmobranch species that have shown a greater negative species-specific physiological response to handling (e.g., *Sphyrnid* species). Overall, very minimal physiological stress was observed in the two treatment groups, which is likely reflected in the plasticity of *T. semifasciata*. Sedation is still often a necessary technique for restraint, depending on the species, the animal’s condition, the type of procedure, and the level of staff training; therefore, the necessity of sedation should be determined prior to performing the procedure. In this study, we provided evidence that manual and chemical restraint as described are comparable techniques that provide little or no difference in stress physiology changes in minimally invasive, rapid examinations.

## 5. Conclusions

The observed increases in lactate, PCO2, and β-HB in both treatment groups likely reflect transient physiological responses to the procedures rather than indicators of sustained or severe stress. Lactate accumulation, for instance, is a common result of anaerobic metabolism, is often seen during brief periods of exertion or physical handling, and typically resolves once the animal returns to a resting state. Similarly, elevated PCO2 levels may indicate temporary respiratory adjustments in response to either the sedation or the restraint procedures, as animals experience short-term alterations in breathing patterns or ventilation. These changes are consistent with the metabolic adjustments observed in other elasmobranch and teleost studies during short-term handling or stress events. While the statistical significance of these changes cannot be overlooked, the magnitudes of these alterations fall within ranges documented in similar studies, suggesting that they represent normal, short-term physiological responses to the handling process. Overall, these transient physiological shifts support our conclusion that both manual restraint and sedation elicit only minimal and temporary stress responses in leopard sharks.

This study is the first to compare parallel handling with the same population of elasmobranchs under two different treatment conditions. The authors encourage future researchers to further investigate and compare techniques for their study species to develop robust evidence for protocol choices.

## Figures and Tables

**Table 1:** Experimental treatment group age class, total length, enclosure location, and sex ratios *of n* = 22 leopard sharks (*Triakis semifasciata*) at the time of selection into manual-restraint or sedation treatment groups.

Age Class	Total Length (cm)	Enclosure	Sex	Treatment Group
Juvenile	61–68	HLD	0.3.0	Manual Restraint
		HLD	1.2.0	Sedation ^a^
Adult	94–160	SAT	2.7.0	Manual Restraint
		SAT	2.5.0	Sedation ^a^

^a^ Tricaine methanesulfonate (MS-222, Syndel, Ferndale, WA, USA) (80 mg·L^−1^); HLD: holding enclosure; SAT: The Sharks of Alcatraz tunnel.

**Table 2:** Means ± SD are listed for each blood plasma i-STAT measurement, energy metabolites, lactate, osmolality, hematocrit, and corticosteroids for the first blood sample (at time 0 min) and at the second blood sample time point (10 min), for the manual-restraint and sedation treatment groups in *n* = 22 leopard sharks (*Triakis semifasciata*).

		Blood Sample Collection Time and Treatment Group		
	Time 0 Reading		10 Min Reading	
Measurement (Units)	Manual Restraint	Sedated	Manual Restraint	Sedated
**i-STAT Blood Gasses**				
pH_TC_	7.68 ± 0.05	7.71 ± 0.05	7.60 ± 0.09 *^+^	7.41 ± 0.075 *^+^
pH	7.37 ± 0.04	7.39 ± 0.04	7.30 ± 0.08	7.13 ± 0.07
PCO_2.TC_ (partial pressure of carbon dioxide; mmHg)	6.35 ± 0.97	5.94 ± 0.74	7.94 ± 2.10 ^+^	9.68 ± 1.84 *^+^
PCO_2_ (partial pressure of carbon dioxide; mmHg)	16.49 ± 2.23	15.13 ± 1.95	18.72 ± 6.30	25.58 ± 5.02
PO_2.TC_ (partial pressure of oxygen; mmHg)	12.7 ± 5.68	16.13 ± 8.06	23.18 ± 18.69	12.22 ± 6.42
PO_2_ (partial pressure of oxygen; mmHg)	56.50 ± 19.65	67.38 ± 22.08	79.00 ± 40.46	54.44 ± 24.44
TCO_2_ (total carbon dioxide; mmol·L^−1^)	15.3 ± 16.77	9.5 ± 0.76	9.45 ± 1.75	9.00 ± 5.45
HCO_3_ (bicarbonate; mmol·L^−1^)	9.43 ± 0.72	9.04 ± 0.60	8.79 ± 1.65	8.34 ± 0.84
BE (base excess/deficit; mmol·L^−1^)	−15.9 ± 0.74	−16.13 ± 0.83	−17.82 ± 1.17 *^+^	−20.67 ± 1.80 *^+^
SO_2_ (oxygen saturation; %)	84.4 ± 11.57	91.00 ± 5.83	83.72 ± 21.69 ^+^	67.45 ± 24.87 *^+^
**Other Meters**				
β-HB (beta-hydroxybutyrate; mg·dL^−1^; STAT-Site^®^)	0.52 ± 0.09	0.55 ± 0.08	0.56 ± 0.11 *	0.55 ± 0.33
Glu (glucose; mg·dL^−1^; Accu-Chek^®^)	82.17 ± 7.22	85.00 ± 8.96	82.08 ± 10.75	86.80 ± 50.44
Lactate (mM; Lactate Scout^®^)	0.47 ± 0.27	0.43 ± 0.34	1.73 ± 0.67 *	3.26 ± 0.75 *
Osmo (osmolarity; mmol·kg^−1^; Osmette^®^ III)	968.42 ± 22.80	1072 ± 324.25	974.75 ± 22.74	992.20 ± 673.17
Hct (hematocrit; %PCV; TRIAC^TM^ Clay Adams^®^)	20.80 ± 3.50	21.40 ± 4.68	21.63 ± 1.89	23.60 ± 4.67
**Enzyme immunoassay (EIA)**				
**Corticosteroids**				
1α-OHB (1α-hydroxycorticosterone; ng·mL^−1^)	13.99 ± 3.46	14.84 ± 4.80	14.52 ± 4.56	17.72 ± 5.04
B (corticosterone; ng·mL^−1^)	0.26 ± 0.47	0.36 ± 0.30	0.36 ± 0.64^+^	1.07 ± 0.20 *^+^

TC indicates that the measurement was temperature-corrected for the pH, PO_2_, and PCO_2_ using Abbot i-STAT Alinity; Heska Corp., Fort Collins, CO, USA; CG8+ cartridge. Where both a TC and a non-TC value are provided, only the TC value was statistically evaluated. * Indicates a statistically significant difference between time point 0 and 10 min within a treatment group. All *p* < 0.05. ^+^ Indicates a statistically significant difference at the 10 min time point between treatment groups. All *p* < 0.05.

## Data Availability

The data presented in this study are available from the corresponding author upon request. The data are not publicly available due to institutional privacy policies.
